# Maximising the translation potential of electrochemical biosensors

**DOI:** 10.1039/d5cc02322j

**Published:** 2025-08-15

**Authors:** Niamh Docherty, Daniel Macdonald, Alisdair Gordon, Alexandra Dobrea, Veerappan Mani, Ying Fu, Susan Pang, Melanie Jimenez, Damion K. Corrigan

**Affiliations:** a Centre for Advanced Measurement Science and Health Translation, Department of Pure and Applied Chemistry, University of Strathclyde Glasgow G1 1XL UK damion.corrigan@strath.ac.uk; b Wolfson Centre, Department of Biomedical Engineering, University of Strathclyde Glasgow G4 0NS UK; c National Measurement Laboratory, LGC, The Priestley Centre 10 Priestley Road Guildford Surrey GU2 7XY UK

## Abstract

Extensive academic attention has been given to showcasing the potential high-level analytical performance of electrochemical and microfluidic diagnostic platforms across a range of target analytes and disease areas. Despite this high volume of research and proof of concept demonstrations for feasible technology platforms, electrochemical biosensors have not yet realised their full commercial potential, given the well-known advantages of low cost, high analytical sensitivity, ease of multiplexing, compatibility with mass manufacturing techniques and seamless connection to smartphones. This is often not because of limitations in analytical performance, but due to challenges in translating laboratory devices into usable, scalable, and accessible systems. Many commercialised point of care (POC) platforms have struggled to integrate effectively into real-world, low-resource clinical environments, underscoring the need for more holistic development strategies. After providing some background on state-of-the-art developments, this article offers a perspective on the major barriers to successful translation for academic research teams through a discussion of the key elements of the biosensor development and translation process. This feature article highlights the importance of the voice of the user, and the iterative research and development process which cycles through stages of innovation, user requirement consideration, analytical performance determination and ensuring the platform is accessible in a POC format. Recent advances in electrode fabrication, 3D printing, and laser ablation empower academic teams to rapidly prototype for practical application. The article intends to serve as a useful guide for those initiating new fundamental electrochemical sensing studies, highlighting recent literature and recommending steps that academic teams can take at the beginning of projects to maximise the chances of future translational success.

## Abstract 2

This article explores the complex technical and development challenges in translating university-based electrochemical biosensor research into market-ready products, and key factors academic teams should consider on that journey.

## Graphical abstract

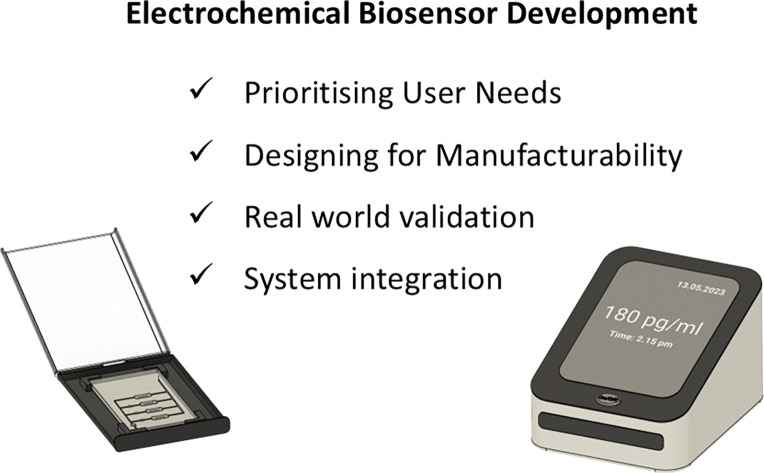

## Introduction

An electrochemical biosensor is an analytical device that combines a biological recognition element (BRE) with an electrochemical transducer to detect target analytes by converting a biological interaction into an electrical signal.^1^ Electrochemical biosensors offer numerous recognised advantages which make them particularly attractive candidates for deployment in the clinical diagnostic space.^2^ These advantages include high sensitivity, improved selectivity using bio-affinity agents, potential for testing multiple targets at once,^3^ seamless integration with a low-cost reader and test strip format, suitability for large-scale manufacturing, and the ability to connect to smartphones and cloud storage for wireless data sharing.^4,5^ Previous perspective papers have expertly summarised the state of affairs of point of care (POC) devices and their current capability.^6^

The clinical diagnostics industry is predicted to reach a value of approximately $108 billion by 2028,^7^ with this covering a wide range of disease areas including but not limited to: diabetes testing (blood glucose, HbA1c, C-peptide *etc.*),^8^ cancer (ctDNA profiling, traditional biopsies *etc.*),^9^ infectious diseases (HIV, tuberculosis, influenza, SARS-CoV-2 *etc*.)^10^ and other clinically important measurements such as prothrombin for blood clotting, C-reactive protein for inflammation and cardiac troponin for acute coronary syndrome.^11^ The diagnostic product format differs across sectors due to varying user needs and settings, which shape the choice of transduction method, such as optical, electrochemical, or piezoelectric.^12^ The most recognised electrochemical platform is the home blood glucose monitor in which patients use a lancet to obtain a drop of blood which is administered onto a capillary filled test strip, to generate a result in approximately 10 seconds. This technology dates back to the 1980s where the principle of using ferrocene as a mediator to shuttle electrons between enzyme and sensor was demonstrated.^13^ The breakthrough accelerated a major research and development effort with home blood glucose monitors eventually leading to widespread use after solving numerous technical challenges along the product development pathway.^14^

The ubiquity, digital readout and user friendliness of glucose meters have attracted researchers to repurpose glucose meters for the detection of non-glucose targets, particularly in low-resource or POC settings.^15^ In recent years, there have been several models demonstrating this for SARS-COV-2 detection that couple the glucose meter with biochemical transduction mechanisms that convert the presence of a target analyte into a measurable glucose signal.^16^ A compelling example of this is a SARS-CoV-2 detection platform that employed a novel fusion protein combining an anti-human IgG antibody with invertase. In the presence of target antibodies, the fusion protein catalysed glucose production, which was subsequently quantified using a commercial glucose meter.^17^ Another transformative development was the integration of the CRISPR/Cas12 system, originally a programmable gene-editing enzyme, into electrochemical biosensors.^18^ CRISPR-based detection systems have been integrated with glucose meters by linking nucleic acid recognition events to enzymatic glucose production, enabling sensitive and specific detection of viral RNA such as SARS-CoV-2.^19,20^ One sensor used CRISPR–Cas12a to recognise SARS-CoV-2 nucleic acids, triggering collateral cleavage that releases invertase from magnetic beads. The invertase then converts sucrose to glucose, which is quantified using a standard glucose meter.^19^ Furthermore, other strategies have combined glucose meter readouts with lateral flow methods,^21^ or aptamer-based systems,^22,23^ transforming these common devices into versatile biosensing tools. These innovations demonstrate how established diagnostic tools can be leveraged for new applications, bridging high-sensitivity molecular biology with familiar POC platforms. However, many of these systems still rely on upstream sample processing steps such as nucleic acid extraction and amplification, which currently limit full integration into a standalone POC system.

Outside of home blood glucose testing, mainstream, centralised clinical laboratory testing still cover the majority of diagnostic capability within healthcare systems. A broad range of diagnostic platforms exist to afford detection of analytes of distinct forms. For instance, bacterial pathogens may be identified by traditional phenotypic microbiology, low levels of bacterial/viral nucleic acids may be detected early by well-established molecular amplification techniques such as polymerase chain reaction (PCR) whereas detection of protein biomarkers or antibody serology may be achieved using familiar immunoassay formats, more specifically chemiluminescence immunoassays and enzyme immunosorbent assays (ELISAs). For readers interested in such technology formats, these are well summarised elsewhere. Between the home blood glucose meter and high-throughput lab systems sits a range of more compact, portable diagnostic technology solutions which are thoroughly discussed in previous literature.^24,25^ While it is possible to achieve high sensitivity analyte detection and robust detection in clinically useful time frames the cost of instrument acquisition and test strips remain high owing to the high degree of complexity of these existing platforms.

**Fig. 1:**
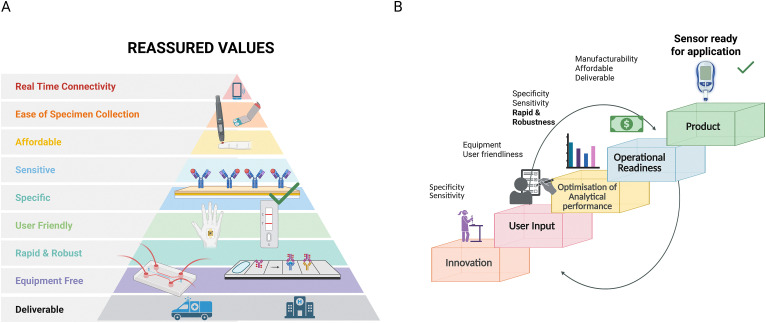
The ideal characteristics of diagnostic tests and a pathway for early stage development towards a biosensor with translational potential. (A) Schematic representing the REASSURED values. (B) User-centred design pathway for medical diagnostics. The schematic outlines a staged progression from innovation to field deployment of diagnostic sensors. Each step incorporates specific design requirements, which align with the recognised REASSUED values. Early stage innovation focuses on technical novelty and feasibility; user feedback shapes usability and functional design; analytical performance ensures diagnostic validity; and implementation addresses affordability, deliverability, and real-time operational readiness. The culmination is a sensor platform that is fully prepared for real-world application. Arrows indicate the importance of iterative feedback loops, particularly between performance and user input. Figure A was reproduced from ref. 26 with permission from Springer Nature, copyright 2019.

**Fig. 2:**
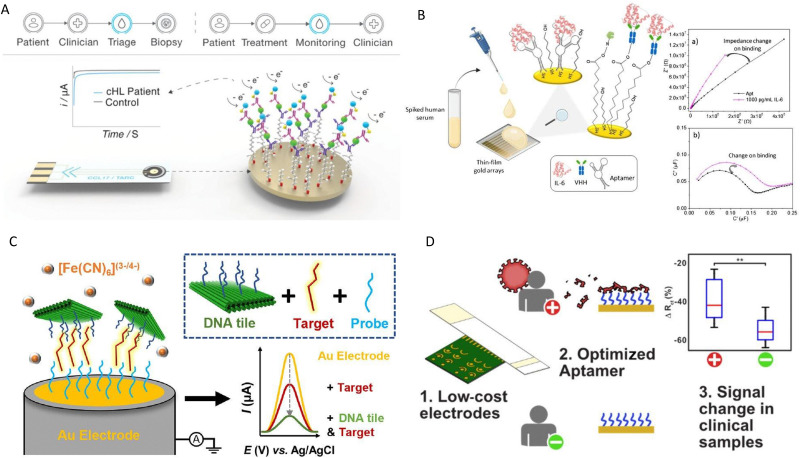
Electrochemical biosensor platforms previously developed by our group. (A) An amperometric CCL17/TARC immunosensor using a thiolated heterobifunctional crosslinker and sandwich immunoassay on an electrode for monitoring of classic Hodgkin Lymphoma. Reproduced from ref. 32 with permission from ACS, copyright 2021. (B) A capacitive electrochemical impedance spectroscopy sensing platform using a nanobody or aptamer for IL-6 detection. Reproduced from ref. 36 with permission from Springer Nature, copyright 2022. (C) Sensor using DNA origami tiles to capture the target and amplify the signal. Reproduced from ref. 30 with permission from ACS, copyright 2023. (D) SARS-COV-2 aptasensors based on electrochemical impedance spectroscopy and low-cost gold electrode substrates. Reproduced from ref. 27 with permission from ACS, copyright 2022. Abbreviations: CHL, classical Hodgkin lymphoma; CCL17/TARC, thymus and activation-regulated chemokine; IL-6, interleukin-6; VHH, variable heavy domain of heavy chain.

Traditionally, pilot and small-scale production facilities were costly, and often outside the scope of academic groups. However, continuous improvements have reduced the cost and size of manufacturing equipment. As a result, in house prototyping and manufacturing are more accessible to academic groups than ever before, allowing them to compete with industry on test strip and instrument cost. It is in this area where a potential advantage may exist for academic groups considering this translational space. To maximise the self sufficiency of academic teams, diagnostic platforms should align with the REASSURED criteria, an acronym created by Land *et al.* (real-time connectivity, ease of specimen collection, affordable, sensitive specific, user-friendly, rapid and robustness, minimal equipment requirements, and deliverable to end-users) (Fig. 1A).^26^ These factors are well known in POC research but often overlooked until after analytical performance is established. At that stage, usability and robustness can be difficult to retrofit, limiting the translation of sensitive electrochemical biosensors. We have identified when in the research pathway each REASSURED criteria should be considered to maximise translational use of academic biosensing platforms (Fig. 1B). By aligning with these considerations throughout academic research projects, the resulting technologies would demonstrate market readiness, regulatory awareness, and user-centred design as well as be clinically relevant. These characteristics together, are the recipe for gaining interest from stakeholders and potential users, enhancing their scope for commercial success. Against the industrial backdrop, research teams are working on biosensor platforms which display ever-increasing sensitivity for key analytes such as blood-based protein biomarkers and low abundance nucleic acid sequences. As a research group consisting of chemists, electrochemists, materials scientists, electrical engineers and biomedical engineers we have recently demonstrated biosensor platforms which err towards simplicity of design and operation (Fig. 2). Recent examples of innovations include: an aptamer modified gold test strip for detection of SARS-CoV-2,^27^ a SARS-CoV-2 biosensor was developed based on glucose test strip manufacturing process where angiotensin-converting enzyme 2 (ACE2) was used as the affinity agent,^28^ 3D printed platforms for the detection of common biological targets such as dopamine, glucose and microbial growth,^29^ use of DNA origami approaches to unlock new levels of sensitivity and capability from DNA modified electrodes,^30,31^ adaptation of common ELISA reagents to aid diagnosis of Hodgkin's Lymphoma,^32^ low-cost surface based amplification of plasmid borne drug resistance genes,^33^ and detection of the same drug resistance genes *via* an ultra-low cost portable potentiostat platform known as ‘SimpleStat’.^34^ Our most recent work involves a low cost thin gold film based amperometric immunosensor, ideal for simple measurements and capable of detection troponin around the 100 pg mL^−1^ range.^35^

To summarise, the strategy of using the academic environment to engineer more simplified biosensor platforms paves the way to cultivate and launch new ventures for exciting future diagnostic products. The academic developmental process may entail significant de-risking by considering the requirements of the regulatory pathway from the beginning, or perhaps even partnering with the regulatory body for endorsement to help attract external investment for product development to ultimately lead to product launch. Noteworthy examples illustrating the improved scope for commercial translation from pilot research platforms include the development of a high signal to noise antibiofouling sensor surface coating,^37,38^ platforms which directly utilise glucose meters to give low-cost and easy to use and simple readout,^15,17^ and direct enzyme engineering approaches that simplify platform designs.^39^ With the advent of rapid prototyping facilities within academic labs, the wide availability of high-quality affinity reagents (antibodies, aptamers, nanobodies, molecularly imprinted polymers), and the ever-increasing ability for academic groups to build liquid handling platforms closely mimicking the industrial capacity and capability By comparison, many large diagnostic companies face high ‘sunk costs’, to establish production facilities, product formats, injection moulding tools, and regulatory approvals, whereas academic teams potentially have an ‘agility’ to their development pathway and a freedom to envisage new product formats rather than work within existing ones.

This article introduces and discusses key factors which academic groups may wish to consider when deciding how far to take a biosensor project along the pathway from concept to early prototype or product. As pointed out above, much of the equipment and technical capability is now available to academic teams such that crucial elements of success can go beyond straightforward technical considerations and shift focus from a blue-sky approach to a more formal, documented approach to sensor development alongside making well informed choices relating to sensor design and eventual production. The remainder of this article will cover what we consider to be crucial factors which should be considered when deciding electrochemical biosensor project structure. Throughout this article cardiac troponin determination for the diagnosis of heart attacks is used as a case study of the dynamic nature of the clinical environment with ever-changing user context and requirements, with the added complexity of capture and detection of low molecular weight targets. The impact of early platform decisions such as sample choice (blood, serum, saliva, urine *etc.*), material choice and usage of microfluidics on translation potential is also discussed.

## Definition of user requirements

Adequate detection limit, high reproducibility and long-term stability are crucial for biosensors. It is equally important that these devices meet the broader needs of the users, patients and potential buyers.^40^ Biosensors developed in academic settings often prioritise sensitivity and specificity, producing sensors that are not developed, verified and validated to the extent required for effective translation for clinical applications. As further work progresses, challenges with robustness, reproducibility, and adaptation issues often emerge, highlighting the importance of early-stage validation and user-centred design.^40^

Ideal POC tests can be used by healthcare workers or patients themselves, and it is assumed that they have limited or no prior laboratory experience. To reduce operator errors, user-friendly devices must be developed that involve quick, straightforward, and minimal steps for the operator.^6^ The importance of each REASSURED criteria varies depending on the user and their surroundings. Acquiring the knowledge to ensure each criterion is met would brief researchers and shape early prototypes. In biosensing, the user context encompasses the physical environment, sample type, its impact on testing, operator experience, resource constraints, and the urgency required for patient safety and management. In clinical settings, the usability and adoption of these devices depend not only on the test operator but also on their placement within the diagnostic pathway and the requirements of first responders, primary care physicians, hospital clinicians, and budget holders.

A user-centred design approach ensures diagnostic tests are practical, accessible and seamlessly integrate into clinical workflows.^41^ One iterative methodology within user centre design that is particularly relevant to point of care testing (POCT) is ‘design thinking’, which enables anticipation of challenges earlier by focusing on user needs, technology feasibility and practical implications.^42^ As such, essential considerations, including regulatory requirements, instrument and test strip manufacture, distribution and storage constraints are accounted for throughout the research and development process.^43^ As a key driver of knowledge generation and innovation, academia can bridge translational gaps by adopting a user-centred approach and integrating elements of an industrial mindset.^44^ Advancements in nanotechnology, machine learning, electrochemical sensing and drug production have been applied to overcome practical challenges such as reagent stability, miniaturisation, sample handling and data interpretation.^40^ These advancements may also be leveraged to tailor diagnostic devices to user needs, improving real-world adoption and impact.^45^

The development of cardiac troponin sensors for acute myocardial infarction (AMI) diagnosis exemplifies the nuanced challenges of biomarker detection and the practical considerations required for end-user implementation. Cardiac troponin is the gold-standard biomarker used to diagnose AMI due to its ubiquitous presence in blood shortly after symptom onset.^46^ However, traditional assays require centralised laboratories, sample transport and trained personnel, leading to diagnostic delays, particularly for patients in rural or low resource areas.^47^ Delays in obtaining troponin results can adversely affect patient care and contribute to emergency department overcrowding, as patients may need tests to be repeated and remain under observation until results are available.

Cardiac troponin is notoriously difficult to detect in blood at its normal level of 0 to 40 pg mL^−1^ range.^48^ Even those presenting with serious myocardial injury, have a median presentation troponin level of around 50 pg mL^−1^.^49^ Compared to many other biomarkers, this represents an exceptionally low detection threshold. While achieving such sensitivity is a major analytical goal, the pursuit of ever-lower limits of detection can risk overshadowing equally critical factors such as assay reproducibility, robustness, and the practical constraints of POC implementation. Many researchers choose cardiac troponin to demonstrate the high sensitivity and specificity of their platforms. However, electrochemical biosensing detection has not been adopted in a true POC setting. Some progress in troponin detection has led to the development of compact benchtop devices capable of ultra-low detection, offering potential improvements in clinical diagnostics. Implementing high-sensitivity cardiac troponin assays at the POC has been associated with reduced emergency department length of stay and improved patient management.^50,51^ However, these devices have yet to see widespread adoption,^52^ partly due to inconsistent effectiveness observed in clinical trials.^52^ There is a general consensus that current POC cardiac troponin devices are effective at ruling out AMI in low risk patients within 2 hours,^53^ though it remains unclear whether the implementation of rapid turnaround tests is beneficial for the timing of revascularisation for AMI patients.^54,55^ Several trials note instances in operator error or lack of coordination and integration into existing diagnostic pathways. The reasons for the uncertainty include the variation in cut-off thresholds for the same analyte across different devices and diagnostic algorithms, thus complicating direct comparisons between central laboratory and POC tests. In addition, clinicians are cautious to rely on POC test results for patient management decisions are not solely based on POC test results, others factors such as clinical risk scores, patient history, and symptom onset must be considered and will ultimately impact turnaround times.^52,56,57^ Multi-centre trials have yielded conflicting outcomes across different centres, highlighting that the effectiveness of POC devices may be compromised by operator error and challenges encountered in busy or resource-limited settings despite their potential for improving patient outcomes if tests are used appropriately.^57^ These findings are not uncommon for trials examining POC adoption in clinical settings. Therefore, earlier consideration of the user and preset diagnostic cut-off values and diagnostic algorithms could minimise the non-concordance observed during POCT validation.

Given the dynamic nature of AMI diagnosis, a universal solution for cardiac troponin remains elusive. Ideally, tests should be functional in different user contexts, such as pre-hospital environments, rural hospitals, and major cardiology clinics in central hospitals. To facilitate their adoption, future troponin biosensor development must prioritise not only analytical sensitivity but also usability, workflow integration and on-site clinical validation. Therefore, successful translation from research to clinical practice requires close collaboration between academia, clinicians and industry to align technological advancements with actual end-user requirements.

## Major barriers to developing successful sensors for practical applications

Exceptional devices continue to emerge from academic labs,^58–61^ underlining the pivotal role of applied sciences in biosensing and POC detection progress. However, several factors limit the translation of these technologies beyond proof-of-concept. On the academic side, heavy teaching loads, short-term funding cycles, and the pressure to publish can deter researchers from investing in long-term, high-risk translational efforts. Funding bodies often favour publication-driven outputs, making it difficult to secure support for in-house manufacturing or scale-up activities. Navigating intellectual property protection and technology transfer processes can discourage progress, especially when institutional infrastructure is not designed with commercialisation in mind. These pressures constrain the time researchers can devote to cross-disciplinary collaboration, clinical engagement, and iterative design, which are crucial steps when developing user-centred, deployable devices.

In parallel, significant technological barriers further complicate the translation from lab to clinic. Many proposed sensor platforms, particularly those based on advanced nanomaterials or novel transduction mechanisms, face unresolved challenges, including non-specific binding, signal instability, limited reproducibility, and sensitivity to complex biological matrices. While performance may appear promising in controlled laboratory conditions, on-site usage often expose issues related to long-term stability, storage conditions, biofouling, and robust calibration across users or sample types. Without addressing these limitations, even technically sophisticated sensors are unlikely to achieve the consistent performance required for successful translation.

Although academic constraints and technological immaturity may appear distinct, they are often interdependent. A lack of sustained funding or interdisciplinary support can prevent necessary optimisation cycles or inhibit engagement with clinicians during early design stages. Encouragingly, there are signs of change, with literature highlighting the value of integrating considerations of manufacturing, deployment, and end-user needs from the outset. As the diagnostics market grows, universities and investors are increasingly recognising the value of commercialising sensor innovations. Shifting funding structures, fostering collaboration, and adopting evaluation metrics that prioritise translatability alongside technical performance may help bridge this divide. When researchers, industry partners, and healthcare providers collaborate from the outset, they can ensure user-centred design is used throughout the development process. Embedding translational thinking at both the researcher and institutional levels will enable device performance to be aligned with practical demands, thereby improving the likelihood of successful real-world deployment.

## Defining the target product profile

A target product profile (TPP) is a critical tool in the development of medical diagnostic technologies, as it provides a clear, structured framework that defines the intended use, performance characteristics, and key attributes of the product. The process of developing a TPP is outlined elsewhere,^62^ but by outlining essential parameters such as the disease or condition to be diagnosed, the target population, desired sensitivity and specificity, and operational requirements (*e.g.*, cost, time to result, and user environment), the TPP ensures alignment among project stakeholders, including researchers, product developers, and eventually regulators and end-users. It guides decision-making throughout the product development lifecycle, from initial design to clinical validation, regulatory approval and commercialisation. Moreover, a well-crafted TPP helps mitigate risks by identifying potential challenges early, ensuring the final product meets clinical needs and market expectations.^63^ The World Health Organisation have a growing list of TPPs in response to emergencies or epidemics. The profiles outline essential product attributes and help accelerate product development by providing clear direction, example of TPPs released already include COVID-19 and bacterial meningitis diagnostic devices.^64^ TPPs vary in length and depth of detail but are customisable to the analyte and user context. One study found that many TPPs neglect cost,^63^ suggesting that cost is not considered until later in the product development pipeline. Implementing TPPs and instilling the REASSURED values by including the cost of goods, testing strip and instrument would be of immense value to academic teams at the project outset. Ultimately, the TPP serves as both a blueprint and a benchmark, fostering the development of effective, user-centred and impactful diagnostic solutions.^65^Table 1 is an example TPP, which academic groups can use to aid planning for biosensor development projects from the laboratory stage to more focused product development.

**Table 1:** Template example of target product profile for point of care IVD test

Category	Requirement
Performance	Qualitative or quantitative: quantitative
	Analytical sensitivity: ≥50% (in a health reference population)
	Specificity: ≥99% (in a reference population)
	Precision: <10% CV at the 99% percentile
	Limit of detection: 1 pg mL^−1^
	Assay range: 1–1000 pg mL^−1^
	Sample type: whole blood/plasma/serum
	Collection tube anticoagulant type: citrate or EDTA
	Sample volume: 30 μL
	Test turnaround time: ≤20 min
	Sample should be applied by fingerprick contact or measurable blood collection micropipette
	±25% of sample size will not affect result

Quality	Inclusion of quality control standards with IVD test kit
	IVD test kit should be able to ship for 1 week in uncontrolled environmental conditions
	IVD test kits should contain an insert with an accurate description of procedure including any possible sources of error, specific storage conditions and expiry dates
	IVD test kit contents should include batch lot numbers and associated mass or volume
	Packaging should be selected to protect against light and moisture

Stability	Shelf life: 12 months before expiry
	Storage conditions: room temperature or refrigerated
	Transportation temperature stability: should be stable under simulated temperature range of −10 °C to 40 °C
	Transportation humidity stability: should be stable under simulated humidity in range of 10% to 80% humidity
	Electrodes should be stable for 1 hour following removal from packaging in ambient conditions

Regulatory and compliance	Meets ISO 13485 standards for medical devices
	Requires FDA approval and CE certification

## Regulatory considerations

The design, development and manufacture of an *in vitro* diagnostic (IVD) device is a highly regulated field. Before an IVD can be placed on the market, it must undergo conformity testing to ensure that it meets the legal requirements for the region where it will be sold.^66^ There are several sets of regulation globally, but the two largest and most important markets are the EU market which is regulated by the European Commission, through the IVDR 2017/746 regulations and the USA market through the Federal Food, Drug, and Cosmetic Act, Chapter V.

Prior to marketing and selling a device, conformity assessment must be carried out by an independent, competent authority for all but the lowest risk devices. This is widely seen as a complex, time-consuming and expensive process.^67^ Key elements of this process include the involvement of a notified body to independently assess the device design, manufacturing and marketing organisation to ensure compliance with safety and performance requirements and assessment of the organisations quality management, risk management and post market surveillance systems.^68^ Although conformity assessment does not lie forefront in the minds of those who are involved in the early stages of device design and development, it is critical to the long-term success of any resulting product.

In addition to the existing regulatory framework, there are emerging challenges surrounding cybersecurity and artificial intelligence (AI). In terms of AI, the primary challenges lie in the safety and performance of generative AI models, as well as patient privacy and data security.^69^ For data security, the threat from malicious actors is omnipresent, and the ever-increasing connectivity for data transmission presents specific cyber security threats.^70,71^ This raises challenges in terms of the certification of medical devices and the standards frameworks, such as those for medical device software will need to evolve to keep pace with technological progress. In terms of IVD device development, this represents a commercial risk that developers need to bear in mind.

## Selection of biorecognition elements

The selection of BREs is a critical step in designing IVD tests, as it directly affects key performance criteria such as sensitivity and specificity. Traditionally, antibodies have been used in IVD development due to their relatively early emergence, as well as their high specificity and affinity for target analytes. After the emergence of antibodies researchers have sought to improve selectivity, specificity, and reproducibility of capture entities while simplifying manufacturing and, ideally, making it more affordable. As a result, several options have come to the forefront, namely antibody derivatives such as fragment-antigen binding units (Fab) and single-chain variable fragments (scFv), nucleic acid-based aptamers, and molecularly imprinted polymers (MIPs). Each of these BREs presents distinct advantages and challenges, particularly when used in POC clinical tests, such as those for cardiac troponin detection. The properties and merits of each of these BREs have been thoroughly reviewed in other papers,^72–75^ and employed in electrochemical detection of cardiac troponin.^76,77^ However, at times the role of the BRE within these systems is not always clearly characterised and justified. In this section, we will emphasise the need for proper consideration of BRE choice and validation before initiating biosensor development. We will compare the utility of various BREs, their use in recent cardiac troponin sensing systems and address the key risks and hurdles that should be considered in early phase development.

Antibodies remain the dominant BRE in biosensing. They are often used in cTnI biosensors as a strong foundation to aid the demonstration of innovative signal amplification techniques such as catalytic and high surface area nanoflowers,^78^ and nanocubes,^79^ to achieve ultra-low detection limits (31 and 9.85 × 10^3^ fg mL^−1^). Another recent example of their use was a platform that utilised thionine-labelled detection antibodies to detect troponin *via* a redox reaction elicited upon antigen–antibody interaction. Furthermore, hybrid biorecognition strategies have been investigated, using dopamine as the signal molecule for a cTnI sensor with an ultralow detection limit a LOD of 0.92 fg mL^−1^.^80^ Despite their performance in ultrasensitive platforms measuring small molecular targets, challenges remain with the traceability, validity of performance, and challenges more directly related to the commercialisation of immunosensors, such as their expensive, timely and complicated production and inherent inter-batch variability.^76^ While some authors have pointed to the inconsistency of commercial antibodies and suggested aptamers as a replacement to reduce resource wastage from underperforming reagents,^81^ we maintain that antibodies remain highly effective. While alternatives such as aptamers and MIPs continue to play a role in diagnostics, *de novo* design could provide a significant edge, yielding more versatile and efficient antibodies. Traditionally, antibodies are raised *via* animal immunisation or antibody library screening, but this may soon change. *De novo* production of antibody-derived recognition elements through rationalised *in silico* design is now possible. Bennet *et al.* have demonstrated the potential of designing antibody VHH domains and scFv units using the RFdiffusion machine learning model.^82^ This rational design approach, validated through cryogenic electron microscopy, marks the first successful confirmation of structurally accurate *de novo* antibodies. This technique enables precise targeting of specific epitopes, simplifying antibody production, reducing costs, and improving binding specificity and affinity. While alternatives like aptamers and MIPs continue to play a role in diagnostics, *de novo* design could provide a significant edge, making antibodies more versatile and efficient.

Antibody fragments, including single-chain variable fragments and fragment antigen-binding units, hold promise by enabling higher packing densities on sensor surfaces and potentially enhancing avidity and sensitivity. However, their application in cTnI detection has not been extensively documented.^83^ One paper demonstrated the use of Fab units to detect HT-2 mycotoxin, which is typically detected *via* a competitive assay due to its small size. The Fab sandwich immunoassay exhibited a 10-fold improvement over the standard competitive assay.^84^ Another compared an IL-6 impedance aptasensor and a nanobody-based sensor, with higher sensitivity achieved by the nanobody approach.^36^ Recent work used time-resolved fluorescence immunoassay, highlighting the importance of site-specific modification of scFv for improved assay performance.^85^ Furthermore, these performance claims require greater empirical validation, and further definition of site-specific immobilisation techniques will be key to maximising their potential.^83^ Immobilising antibody fragments has similar hurdles to antibody immobilisation, including a reduction in binding affinity and specificity due to improper orientation, conformational changes, or steric hindrance, which results in impaired electrochemical signal generation.^77^ Additionally, the stability of antibodies over prolonged storage or under fluctuating environmental conditions is a concern in POC applications given that consistent performance across multiple assays is essential.^86^

**Fig. 3:**
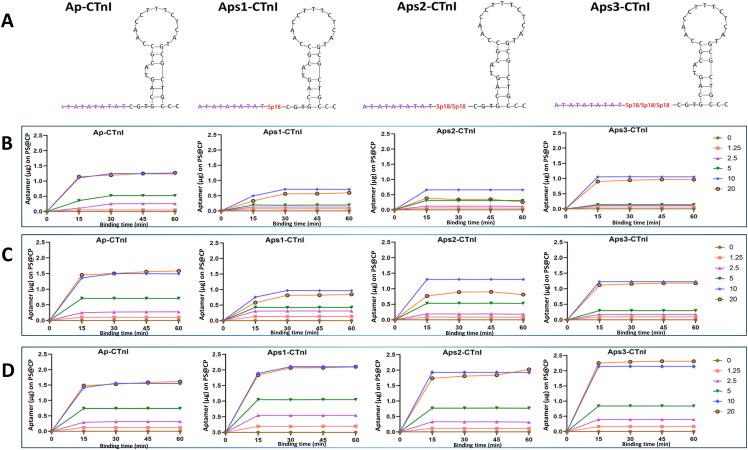
Design and screening of cTnI aptamers (Ap-CTnI, Aps1-CTnI, Aps2-CTnI, Aps3-CTnI) for optimal binding conditions to sensor substrate PEDOT:SWCNTs conducting paper. (A) The structure of the cTnI aptamer after modification, the anchor domain was modified (purple), and 18-atom hexa-ethylene glycol spacers (Sp18, red) were added between the anchor sequence and the capture domain. (B)–(D) The optimal folding temperature, adsorption time, and concentration of the four modified cTnI aptamers were screened on PS@CP. Abbreviations: PS@CP, PEDOT:SWCNTs conducting paper; PEDOT, poly(3,4-ethylenedioxythiophene); SWCNTs, single-walled carbon nanotubes. Reproduced from ref. 91 with permission from RSC Published, copyright 2025.

Aptamers are of increasing popularity for cTnI electrochemical detection due to their high affinity, ease of synthesis, and stability. Well-established nucleic acid synthesis technologies lend themselves to reduced production time and cost compared with antibody synthesis.^87^ They are frequently applied to enhance assay sensitivity by combining their high affinity with innovative signal amplification approaches, such as magnetic particles and the addition of catalytic metal–organic frameworks to reach impressive detection limits (0.31 fg mL^−1^).^88^ Signal enhancement has also been demonstrated using polymerisation-mediated silver nanoparticle deposition,^89^ hybridisation chain reactions for amplification,^90^ and advanced surface modifications that minimise steric hindrance and optimise charge transfer efficiency, with LODs in the fg mL^−1^ range consistently achieved. Label-free aptasensors have also been explored, demonstrating highly sensitive detection through ion barrier effects and redox reactions, with a reported cTnI LOD of 2.03 fg mL^−1^.^60^ In addition to yielding highly sensitive detection devices, aptamers are attractive POCT candidates due to the low cost and accessible synthesis methods available. However, aptamers face challenges in clinical applications due to inconsistent binding affinity and performance across varying assay conditions.^75^ Challenges regarding their structural stability have prompted investigations into optimal annealing conditions and spacer inclusion to preserve their functionality during immobilisation. In this regard, Xie *et al.* applied a rational approach to delineating these complex factors and identified that increased spacer length and a staggered, gradual annealing approach maximised the detection sensitivity achieved (Fig. 3).^91^ Aptamers have also been identified to exhibit impaired structural stability, particularly in complex biological matrices, where they may degrade or lose their conformational integrity.^88^ Furthermore, when aptamers are functionalised on electrode surfaces, their binding conformation can be altered, resulting in reduced specificity and weaker affinity.^90^ Aptamer-based sensors may also be prone to non-specific interactions or matrix interference, potentially generating false positives and impacting assay sensitivity.^58^

MIPs are robust, cost-effective, and highly stable recognition units for cTnI detection. MIPs have been utilised to generate high-affinity recognition sites through polymerisation around a cTnI template, with surface modifications like graphene quantum dots and gold nanoparticles enhancing sensitivity, resulting in LODs as low as 10 pg mL^−1^.^59^ Single-step MIP-based strategies that rely on signal “switch-off” mechanisms have been demonstrated in both buffer and serum matrices, achieving LODs in the low pg mL^−1^ range.^92^ Dual recognition platforms incorporating MIPs with antibodies or aptamers have also been explored to further enhance sensitivity and allow for multiplexed electrochemiluminescence biomarker detection.^93^ Research has confirmed that these low-cost biorecognition elements can be synthesised with relative ease while ensuring impressive performance.^94^ However, issues such as batch-to-batch variability, binding site heterogeneity, template removal, and sensitivity will need to be refined before these biomimetic reagents can be incorporated into commercial devices.^95^ When applied to electrode surfaces, MIPs may exhibit inconsistent rebinding capacity, leading to decreased reproducibility and sensitivity.^92^ MIPs also face durability challenges under clinical conditions where pH, temperature, and ionic strength fluctuations may affect their structural integrity and binding performance.^95^ Overcoming these hurdles could enable broader application of MIPs as sensing elements, leveraging their chemical stability, low cost and accessibility.

While acknowledging that time and funding constraints can lead to premature BRE decision-making, it is essential to explore viable bioreceptor options when developing electrochemical assays, particularly in small-scale settings. We have compiled information from seminal papers and more recent discussions on the efficacy and practicality of standard bioreceptor options as well as their associated risks, which often negatively impact the likelihood of translation or the performance of the platforms outside a controlled laboratory environment (Table 2). From there, we recommend researchers evaluate several BREs to find a superior BRE for their intended use. For example, antibodies can be screened in an ELISA format before moving on to electrochemical sensor development. For any biorecognition element to be successfully translated into clinical applications, stability, durability, and regulatory compliance must be addressed.^96^ Clinical diagnostics require reagents that maintain performance over extended periods and under varied storage conditions, which is not yet readily achievable for current BREs.^97^ Moreover, regulatory frameworks require rigorous validation to ensure reproducibility, accuracy, and safety, adding complexity to commercialisation efforts.^86,98^

**Table 2:** Selection criteria that may be applied to assess the risk associated with different types of biorecognition unit. Abbreviations: EDC, NHS, SELEX

Selection criteria	Antibodies (monoclonal and polyclonal)	Antibody derivatives (Fab's/scFv)	Nucleic acid analogues (aptamers)	Synthetic biomimetics (MIPS)
Quality	Well-established commercial manufacturers with quality-controlled production lines. Quality and consistency are largely defined by manufacturer's production standards^72^	Increasing availability of antibody derivatives from quality-controlled commercial suppliers	Consistent quality based upon well-defined chemical solid-phase nucleic acid synthesis procedures following SELEX selection^73^	Very few commercial sources and lack of clarity of standards and quality control^74^
Performance	Subject to empirical validation, maximised when sourced from “trusted” suppliers with recognised quality accreditation. Requires internal validation after sourcing^72,97^	Comparative performance to antibodies small size allows for higher packing densities enhancing sensitivity^75^	Comparative or higher binding affinities than antibodies,^99^ strong potential for low molar mass analytes,^73^ and small size enables greater surface packing density^99^	Promising for low molar mass analytes, potentially limited to nanomolar detection range^99^
Consistency	Excellent consistency thanks to established methods, polyclonal antibodies are more vulnerable to batch-to-batch variation^81^	Fair consistency – variability may arise from expression vector selection, challenges include endotoxin production, protein misfolding, cell line instability, low yields, and glycosylation, variability compromises stability and purity^97^	High consistency due to ease of standardised nucleic acid synthesis technologies^81^	Batch-to-batch variability^99^ due to non-specific binding to the target molecule & heterogeneity of target binding sites during production^74^
Stability	Fair but prone to proteolysis, denaturation or microbial contamination, so requires cold chain storage^72^	Satisfactory but prone to proteolysis, which may limit industrial application.^100^ ScFv is less stable, shorter half-life compared to Abs^101^	Highly stable due to ease of lyophilisation and no need for cold chain storage, but vulnerable to nuclease degradation in solution unless modified^73^	Can be stored indefinitely and relatively stable in various temperature and pH conditions, resistant to proteases and nucleases^74^
Production time	3 to 6 months^86^	3 to 6 months – Fab generation from precursor antibody^102^	2–8 weeks^73^	Less than 1 month^86^
Production cost	Expensive due to the requirement for animal inoculation and hybridoma cell production^73^	Precursor antibody generation and recombinant expression technology is expensive. Chemical Fab’ generation from a whole antibody is low cost and simple^102^	SELEX process is expensive,^102^ but new technology^73^ is typically cheaper and more readily scalable^96^	Low cost of bulk synthesis may lend itself well to decentralised production and application^99^
Ease of chemical modification	Limited but robust modifications, *e.g.* thiolation, biotinylation and EDC/NHS^75^	Wide range of linkers available for Fab C-terminal thiol,^102^ Positively charged amino acids, poly-histidine tags, biotinylation or terminal cysteines.^75^ can be added to ScFv *via* recombinant expression	Easily modified using standard chemistries, including thiolation and amination,^103,104^ with high degree of control over site specific modifications to sugar, backbone and base moieties^73^	Post-imprinting modification possible to introduce new functional groups such as thiols, carboxyls and reversible disulphide or imine bonds providing versatility^105^

The behaviour and performance of BREs can vary depending on the substrate it absorbs or is attached to, causing steric hindrance, diminished binding affinity, or altered conformational stability, potentially causing signal loss, non-specific binding or reduced sensitivity.^78^ Additionally, the electrochemical environment may cause oxidative stress or degrade sensitive biomolecules, necessitating careful selection of immobilisation chemistries and protective surface modifications.^102^ Thus, it is vital to have a good understanding of the performance of the BRE in regard to binding to the intended target. BRE validation is necessary, even for commercially sourced components, under conditions that reflect routine operational conditions, *i.e.* complex samples. Assessing signal-to-noise ratios and potential interference at this stage helps ensure relevance to clinical or field conditions. Once validated, these BREs should be rapidly integrated into biosensor platforms to further test performance under realistic use scenarios.

Ensuring transparency in validation data across all BREs will enhance reproducibility, performance, and confidence in assay development while addressing the challenges of stability, electrochemical compatibility, and regulatory compliance necessary for successful clinical translation.^100^ Researchers often presume the suitability of these elements for their intended applications. However, significant concerns have emerged regarding the effectiveness and consistency of commercially available antibodies. Studies reveal that many antibodies do not meet quality control validation standards; for example, a 2008 study indicated that only 49% of 5436 antibodies from 51 suppliers passed a fundamental immunohistochemistry validation test.^99^ The antibody characterisation through Open Science (YCharOS) initiative aims to validate antibody performance and enable transparency in antibody selection independently.^81^ This initiative has drawn attention to the issue of ineffective antibodies, contributing to the “reproducibility crisis”,^97^ with estimates suggesting that 20–30% of published data relies on antibodies that do not bind effectively to their intended targets.^100^ Establishing a universally accepted method for antibody validation is complex, with various techniques proposed, such as western blot, immunoprecipitation, and immunohistochemistry.^96,102^ However, these methods do not ensure that antibodies will maintain functionality on biosensor surfaces, placing the responsibility of screening and validating these elements on researchers. While traditional validation techniques are valuable, in-house screening, like the checkerboard approach,^106^ is essential for optimising assay performance in complex samples. Researchers are also encouraged to utilise databases like Antibodypedia, CiteAb, and BenchSci for validation data, facilitating informed antibody selection.^103–105^ As interest in biorecognition elements like antibody fragments, aptamers, and MIPs increases, expanding these resources to include such elements will support the development of reliable diagnostic tests.

To summarise, researchers have an expanding array of BREs to meet product and user requirements, including sensitivity and specificity. The introduction of low-quality or poorly performing reagents contributes to the irreproducibility crisis. However, internal validation is often required to ensure biorecognition elements perform in the intended use conditions. Issues with antibodies typically arise from the traceability and validation framework of commercial production. Transparent publication of validation data and a unified framework would help ensure consistent quality and validation, saving researchers countless hours by enabling them to make informed decisions. While aptamers and MIPs offer promising alternatives, their inherent challenges have prevented them from fully replacing antibodies. Continued advances in antibody production, including the use of fragments and *in silico* design, position antibodies as the most reliable biorecognition elements for electrochemical sensing. Clear validation of alternative BREs and emerging technologies will provide researchers with the flexibility to select the most appropriate platform based on specific application requirements. Ensuring transparency in validation data across all biorecognition elements will empower researchers to tackle BRE-related issues and create REASSURED value-aligned platforms.

## Electrochemical sensing: modalities, innovations, and impact

Electrochemical biosensors have made significant progress in the detection and quantification of cardiac biomarkers. These methods rely on interfacial changes occurring during antigen–antibody interactions and the transduction of these events into electrochemical signals to detect low concentrations of target analytes. Electroanalytical techniques such as differential pulse voltammetry (DPV), square wave voltammetry (SWV), amperometry, and electrochemical impedance spectroscopy (EIS) are used.^3,107^ Many cardiac biomarker electrochemical biosensors follow a familiar signal production approach to other sensors presented in the literature, involving enzyme labelling or external redox mediators as the signal-revealing mechanism.^108^ This section will discuss how these electrochemical measurements study the biorecognition element-target interaction and their applicability to real-world diagnostics.

Amperometry is the most successful electrochemical technique in terms of translation from academia to industry, conceived originally as a glucose sensor.^109^ The glucose sensor industry has since fuelled many technological advancements. It enjoys regulatory acceptance and offers advantages such as simple electronics, robustness, reproducibility, user-friendliness, fast response times, and ease of data interpretation.^110^ Amperometry is a highly preferred candidate for sample-to-answer sensors since non-technical personnel can perform the assay with basic training, making it a robust choice for commercial applications.^111^ DPV and SWV are particularly valued for their ability to eliminate non-faradaic current signals and provide high sensitivity compared to amperometry. Commercial environmental sensing applications, including heavy metal detection, utilise these techniques.^112^ However, DPV and SWV-based sensors are less commonly adopted for commercial clinical diagnostic tests. This limited adoption may be due to the need for more advanced circuitry and signal processing.

EIS holds promise as a label-free electrochemical measurement, with many examples of very low detection limits across the literature.^61,113–116^ While EIS is well-regarded in academic research, its commercial potential remains underdeveloped. Barriers to commercialisation in handheld electrochemical readers include difficulties with measurement reliability, reproducibility, and complexity in data interpretation. Since EIS platforms are not routinely used, there is a lack of standardisation and calibration procedures, as well as the absence of clear regulatory standards, which leaves developers uncertain about expected product qualities and boundaries.^117^ Thus, while amperometry remains the most widely accepted technique at the industrial scale in the context of cardiac sensors, there is a significant gap for technological advancements in DPV, SWV, and EIS to translate their reported excellent merits into commercial success. There remains a significant gap for methods like DPV, SWV, and EIS to translate their strong analytical performance into robust, regulated commercial platforms. One solution is to incorporate electrical engineering and commercial technology development expertise from the outset of a research project to drive the creation of complete systems, ensuring a balanced focus beyond just sensitivity and detection limits.

**Fig. 4:**
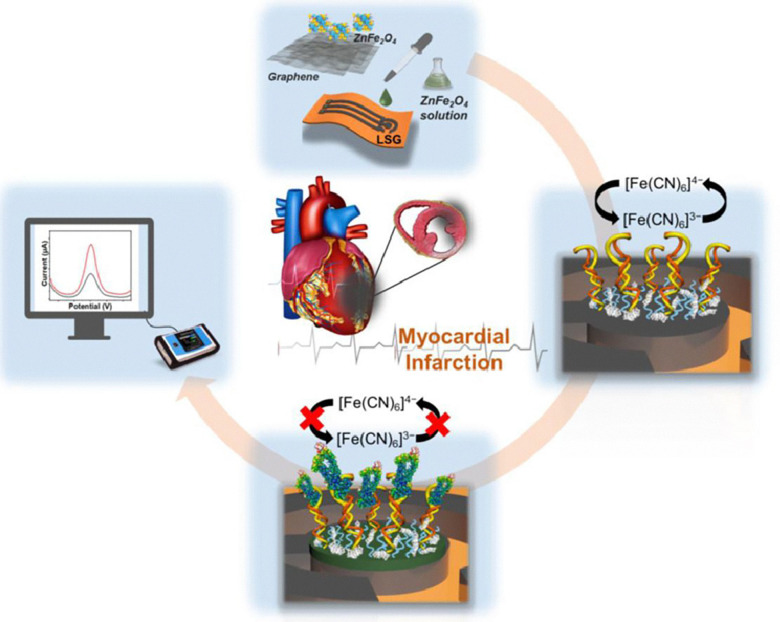
Schematic overview of aptasensors. This example shows a laser-scribed graphene electrochemical aptasensor, modified with metal oxides for sensitive AMI screening. Reproduced from ref. 118 with permission from Elsevier, copyright 2021.

In response to these challenges and the demand for improved diagnostic tools, there has been a growing interest in electrochemical aptasensors for cardiac troponin detection. These platforms leverage electroanalytical techniques—including EIS, DPV, and SWV—to monitor changes in interfacial charge transport that occur upon successful binding of troponin to a surface-immobilised aptamer (Fig. 4).^118^ The development of electrochemical aptasensors is fuelled by the established aptamer sequences for cardiac troponin and the commercial availability of thiolated cardiac aptamers that can be easily immobilised on the electrode surface *via* gold–thiol surface chemistries.^119^ Compared to immunosensors, aptasensors offer simpler electrode fabrication processes. Measurements typically involve monitoring the redox signatures of mediators such as the ferricyanide/ferrocyanide system. Over the last decade, a surge of startup companies developing electrochemical aptasensor technologies emerged, suggesting a strong potential for the commercial realisation of aptasensor technology.

The demand for POCT devices is rising, particularly for cardiovascular diseases which continue to increase in prevalence and require continuous monitoring at the bedside, in ambulances and emergency care settings, and at home, for instance, during post-heart attack recovery.^120,121^ The portable nature and user-friendly operation of electrochemical measurements, combined with their low cost, make electrochemical biosensors highly desirable for POC testing. The key driving factor in electrochemical technologies is its direct connection with the electronics. The major components of electrochemical instruments are based on electronic components and circuits. Consumer electronics is growing at an unprecedented speed in the directions of miniaturisation, flexible electronics, and portability. Such growth is acting as a catalyst in the development of portable electrochemical devices.

Smartphone integration and miniaturised potentiostats make electrochemical measurements relevant for research translations.^5,122^ Walaa *et al.* recently demonstrated the integration of disposable laser-scribed graphene electrodes, miniaturised potentiostat and smartphone for detecting cardiac troponins at physiologically relevant concentrations. Such integrated devices can detect cTnI levels as low as 0.01 pg mL^−1^ when coupled with nanomaterials.^105^ The advantage academia has over industry in these cases is that universities are a hive of experts in a multitude of disciplines, ripe for the picking to create a collaborative and multi-disciplinary team of electroanalytical chemists, electrical engineers, mechanical engineers, computer scientists, and clinicians. Nevertheless, industrial partners can facilitate the transition from academic research into translational research and development.

Electrochemical instruments offer a key advantage by directly measuring signals, unlike optical methods that rely on external lamps to convert analyte interactions into digital signals. They are highly compatible with raw biological samples, like whole blood for glucose detection, making them superior to alternative transduction methods. These benefits have yet to be fully realised in robust POC troponin tests, primarily due to challenges with biofouling.

Biofouling, the non-specific adsorption of biomolecules such as proteins, cells, and lipids onto sensor surfaces, remains a major challenge in electrochemical biosensing. A significant portion of the literature on cardiac troponin electrochemical biosensors includes results based on diluted serum samples to minimise the effect of these biomolecules on sensitivity or specificity. Due to the persistent challenges posed by biofouling, researchers often employ workarounds. Namely, diluting biological samples to reduce matrix complexity, limiting sensor use to single measurements, or conducting only short-term studies to minimise performance loss. Stability studies are often limited to short durations, partly because biofouling accelerates sensor degradation in complex biological samples. In addition to biofouling, other factors such as electrode material degradation and the instability of biorecognition elements contribute to performance decline, making it challenging to demonstrate long-term functionality. Although these amendments allow platforms to achieve clinically relevant performance, they ultimately limit the evidence for translational use.

**Fig. 5:**
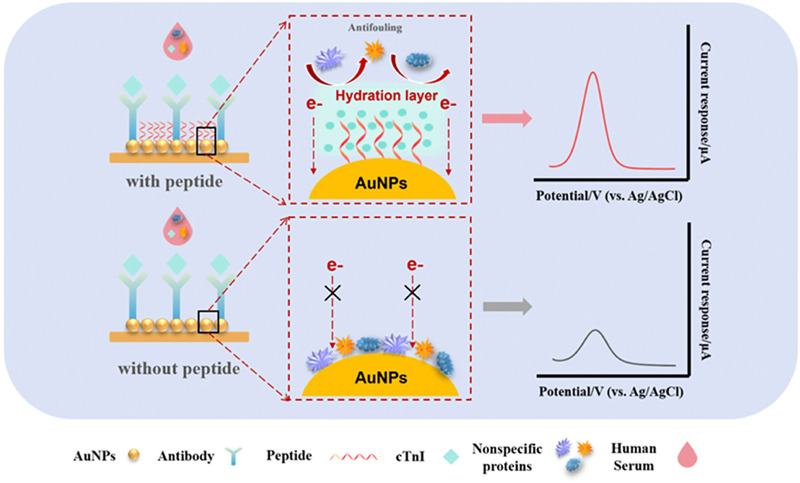
Example of how peptides can introduce a hydration layer, preventing biofouling on an electrode surface. Schematic mechanism of the electrochemical immunosensor for cTnI detection based on CEAK16 peptide. Abbreviations: cTnI, cardiac troponin I; AuNPs, gold nanoparticles. Reproduced from ref. 125 with permission from publisher Elsevier, copyright 2024.

Researchers have proposed various antifouling surface modifications to enable the reliable detection of biomarkers in blood samples over time. These tactics include PEGylation, zwitterionic polymers,^123^ hydrogels, and biomimetic surface engineering.^124^ Effective antifouling designs rely on three primary mechanisms: creating a hydration layer to establish a physical and energetic barrier, using steric hindrance to block molecular access, and employing electrostatic repulsion to deter similarly charged species. These strategies help maintain sensor sensitivity and specificity in complex biological environments. For example, an antifouling cTnI electrochemical immunosensor utilises a vertically aligned layer of amphiphilic CEAK16 peptides combined with gold nanoparticles (CEAK16@AuNPs) to form a stable hydration layer through the hydrophilic amino acids present in the peptide structure. This hydration layer significantly minimises non-specific adsorption in human serum and preserves over 80% of the sensor's electrochemical activity after 20 days, demonstrating strong long-term antifouling performance (Fig. 5).^125^ Apart from addressing antifouling directly, researchers often rely on pre-processed serum samples to reduce matrix and biofouling effects. Advancements in miniaturisation and integration may enable effective sample processing within the electrochemical biosensing platform, which would be necessary for POC suitability. Notably, the stability of many reported cardiac troponin ECBs has only been tested for 30 days, which is insufficient to prove their translation potential.

A key focus in exploring antifouling methods is the feasibility of incorporating an antifouling step into the manufacturing process. Timilsina *et al.* have published numerous papers discussing biofouling strategies, including an ultrafast *in situ* antifouling coating for electrochemical sensors that can be applied through simple dip-coating followed by rapid on-chip heating. This coating consists of cross-linked bovine serum albumin infused with conductive, pentaamine-functionalised graphene particles, forming a hydrophilic, protein-resistant layer that dramatically reduces non-specific adsorption. This approach enables high conductivity, sensitivity, and selectivity for diagnostic applications, demonstrating stable performance for up to 9 weeks in undiluted biological samples and at least 20 weeks of storage stability at room temperature.^126^ Their research further showed that the same antifouling nanocomposite coating can be used for the simultaneous detection of four biomarkers including cTnI.^127^ While some antifouling strategies demonstrate promise, integrating them into scalable manufacturing processes poses challenges. A key barrier to commercialisation remains the need to produce sensors with sufficient stability for long-term storage and repeated use.^128^

One of the key challenges in the electrochemical biosensors is the integration of redox label with the analyte-sensor binding event. Still, electrochemical immunosensors heavily depend on external mediators and enzymatic labels such as HRP or alkaline phosphatase.^107,108^ For instance, commercial troponin assays depend on an alkaline phosphatase-based sandwich immunoassay.^109^ These tagged assay systems provide excellent signal amplification and detection sensitivity. However, the use of redox reporters such as 3,3′,5,5′-tetramethylbenzidine (TMB), methylene blue, ferrocene, and benzoquinone can have a detrimental effect on assay performance with surface fouling and passivation, oxidation/reduction signal drift over time, non-specificity or variation in signal intensity over time.^129^ Research translation could perhaps be directed to strategise the development of robust EIS based electrochemical readers for electrochemical immunosensors.

As the POC and biosensing landscape expands, referees for many journals publishing prototypes of POC devices expect authors to include at least some demonstration using practical samples. To meet this criterion, researchers often simulate practical conditions by spiking target analytes into biological fluids such as human serum or urine and then constructing calibration curves. However, such mock biological fluids have some limitations; it is unknown if assay performance would withstand a more practical sample, such as diluted or preprepared bodily fluids. While this approach has become common practice, the benchmark could perhaps be raised further by introducing a minimum requirement for at least one clinical validation or practical test using whole samples when showcasing a POC platform. This would require researchers to examine biofouling and non-specific adsorption.

Academic publishing has a prevailing emphasis on positive outcomes, which often leads to reduced visibility for negative or null results. Consequently, research papers frequently highlight successful findings, while setbacks, failed experiments, or methodological challenges may receive less attention. Yet, valuable insights and learning opportunities are often found within these less-celebrated aspects of research. To fully leverage the value of earlier academic efforts, a shift in academic culture is necessary, whereby negative results receive equal attention and are openly discussed by academics and industrial partners. This will lead to a greater understanding of the limitations encountered in the electrochemical immunoassay field, allowing new academics to focus on the most relevant research directions.

For research groups focused on developing next-generation biosensing platforms and aiming to translate their advances into tangible product formats, it is essential to reflect on the practical and conceptual challenges that surround electrochemical biosensor development. These have been discussed in this section and our perspective on these challenges is summarised below in Table 3. Our intention is for the questions in Table 3 to serve as a checklist for teams when considering which stage to advance their project to and what practical demonstrations can be made to showcase the performance level of their platform.

**Table 3:** A list of important conceptual questions for researchers seeking to develop and report electrochemical biosensor platform performance

Key questions on translation of electrochemical biosensor methodologies
(1) Is a demonstration of a reproducible measurement at clinically significant levels in a relevant sample matrix more important than a demonstration of exceptional analytical sensitivity in buffer samples?
(2) Are buffer sample measurements only really applicable to a demonstration of a novel sensing modality?
(3) Has any stability/dry down testing been carried out with the reported sensor?
(4) What constitutes a suitable number of replicates to showcase the capability of the developed biosensor platform?
(5) Have all relevant sample interferents been accounted for in the testing regime?
(6) Have the needs of the end user been considered?
(7) Are the receptors, surface chemistries, electrode materials compatible with end scale manufacture?

## Selection of sensor and associated materials

In recent years, the field of POC has advanced significantly, especially with the development of novel sensors and materials. Modern electrode technology yields a range of electrode materials, including screen-printed carbon electrodes, screen printed gold electrodes (SPGEs), and printed graphene electrodes. The primary objective has been to enhance sensitivity, reduce detection limits, improve reliability, increase manufacturing simplicity, and lower material costs. Various materials have been explored in academic research as the basis for cheap, sensitive sensors, with further research put towards the augmentation of these electrodes for improved functionality.

Already a well-characterised and widely used material for sensors, SPGEs have been employed in academic research and industrial applications for some time. Efforts in recent years have focused on enhancing the electrochemical performance and functionality of SPGEs, including the development of thiolated aptamer-based sensors for detecting biomarkers such as thrombin, a coagulation-related protein with relevance in various diseases including Alzheimer's.^127^ In this approach, an ultrasensitive, reagentless aptasensor was constructed using a hemin-G-quadruplex DNAzyme on SPGEs, offering promising analytical performance under laboratory conditions. However, despite improved sensitivity and simplified assay format, challenges remained-such as limited operational stability (∼3 days), susceptibility to biofouling, and weaker thiol-gold interactions on printed electrodes compared to solid gold-ultimately restricting the sensor's robustness for real-world deployment, particularly in terms of commercial shelf-life and shipping resilience.^130^ An alternative gold modification method demonstrated by Kashefi-Kheyrabadi *et al.* showed that SPGE's electroplated with nanostructured gold could be used to create a high sensitivity, cheap and mass produced electrochemical SARS-CoV-2 RNA sensor, demonstrating clinically relevant detection limits of 2.5 and 4.5 ag μL^−1^.^131^ In a similar manner, Kusnin *et al.* used a combination of depositing copper nanowires and backfilling with gold nanoparticles to increase the molecular binding area of their SPGE 2.3-fold, improving electrochemical activity on the surface of the electrode.^132^ These improvements develop the use of gold as a sensor material and continue to prove its competitiveness in the field of POCT sensors.

Graphene, whether through screen printing, dropping or laser inducing is often used in sensor development. Graphene is already in industrial use for sensors, while academic research has combined graphene with other materials to improve its flexibility, binding affinities, and sensitivity in POCT. For example, Kakkar *et al.* functionalised screen printed graphene (SPG) electrodes with aptamer conjugated gold nanoparticles to create a cTnI sensor achieving a range of 0.001–1000 pg mL^−1^, demonstrating sensitivity exceeding most commercially available products.^133^ However, there is some weight behind the argument that the sensitivity of this assay far exceeds the clinical relevance of cTnI. The assay being validated using clinical samples does provide valuable real-world results when considering this platform for the adoption into a commercial product. Graphene has also been utilised as a material in sensor development through combination with paper assays, as illustrated by the amperometric hydrogen peroxide sensor developed by Sun *et al.*^134^ Graphene oxide was dropped onto cellulose paper and further modified through coating with nanoporous gold, then subsequently with platinum and palladium nanoparticles. While this sensor demonstrated good stability times of over 30 days, the lengthy and complex process of manufacturing would be too laborious for commercial production. Another recent interesting combination has been provided by Lin *et al.*, through the addition of hydrogel to screen printed carbon electrodes in order to make a wearable electrochemical glucose sensor.^135^ The hydrogel facilitates the uptake of sweat from the patient, serving as the matrix for the assay and proving to be a suitable material for wearable biosensors.

Laser induced graphene (LIG), typically on polyimide film has been utilised for many years due to being cheap, scalable, and highly amenable to prototype as it is a one-step process with relatively cheap machinery (low power laser scriber), consumables (PI film), and facilities (no clean-room needed). LIG has been utilised in various sensor applications, including the sub-dollar digital microfluidics platform.^136^ A recent paper by Liu *et al.* focussed on demonstrating that LIG platforms can be produced cheaply and simply while retaining competitive performance, which is a compelling endorsement for their use in industrial settings. Furthermore, LIG has shown significant potential for enhancement, particularly when modified with platinum nanoparticles, which improve its catalytic properties, signal strength and detection sensitivity. Coating the LIG reference electrode with Ag/AgCl in a fully integrated system has yielded promising initial results for a cTnI POC test, achieving a detection limit of 45.33 pg mL^−1^ and a quantification limit of 151.10 pg mL^−1^.^137,138^ Another interesting development was the use of metal stencils in LIG production, which gave rise to increased resolution and reliability while allowing more intricate LIG patterns to be created due to the increased accuracy to define the area of PI film exposed to laser treatment.^139^ This simple application of reusable stencils is ideal for both research and industry adoption due to its low cost and clear advantages.

## Consideration of microfluidics and on platform sample separation

Microfluidics and *in situ* sample separation have become increasingly prevalent in both academic research and commercial medical products, in a coordinated drive to reduce reliance on large and expensive equipment and reduce patient waiting time for results. Both sectors aid the other in this aspect, with industry using academic research findings to improve or innovate products and academia gaining funding for relevant research due to the commercial success of said products. However, each sector has its own strengths and weaknesses.

While industry usually has the funding and space for large-scale manufacturing, academia can rapidly prototype new designs, especially using methods like 3D printing and soft lithography, which allow researchers to test iterations quickly.^140^ For example, Zhou *et al.* recently demonstrated the use of a simple novel handheld centrifuge system for separating blood and plasma, coupled with a smartphone for measurement analysis to reduce their dependency on larger, pricier equipment.^141^ In addition to prototyping capabilities, academia has more freedom to pursue high-risk, high-reward projects without the immediate pressure of market constraints. This enables research into areas such as passive separation, including sedimentation, which has shown promising results in blood plasma separation efficiency. However, it requires significantly longer processing times (∼8 min) compared to commercially available powered separation techniques.^140^ The flexibility to pursue riskier projects also allows for creative approaches and breakthroughs that may not initially seem commercially viable but could lead to transformative technologies, such as the hand-powered centrifugal disc developed by Kuo *et al.* for use in urinalysis.^142^ This device miniaturises the equipment typically used for this process while maintaining a high separation efficiency and integrating the sample handling with a combined sediment and chemical strip test. While the 10 min test runtime is appealing to prospective investors, the hand-operated nature of the device would be a drawback, as industry preferences typically lean towards automated processes that enhance user-friendliness and reduce the need for specialised training. Therefore, fully automated systems, such as the multiplexed rotary valve system developed by Chen *et al.*, that require more interdisciplinary work in instrumentation and fabrication are perhaps more likely to be approved for translation to a commercial product if industrial partners are involved.^143^ Research into the underlying principles of microfluidics for sample separation holds significant value for industry and potential funding opportunities, especially if novel enough for patent consideration.^144^ Li, An, and Jeong demonstrated improved mixing efficiency by adding 3D obstructions to Tesla mixers, while Maurya *et al.* reviewed the effectiveness of various microfluidic geometries, such as curvature, T-junctions, and constriction–expansion, providing valuable insights for future research and product development.^144,145^

Another aspect to take into consideration when attempting to translate academic research to a commercial product is scalability. While academia has access to high-precision instruments for limited production runs, scaling the processes up usually presents significant difficulties. As those in industry usually apply considerations for scalability from very early stages in development, they often make use of simpler, more easily applied discoveries and technologies, such as the use of separation filters and capillary action to gain high blood plasma separation efficiency in under 2 minutes by Brakewood *et al.*^146^ This device would be appealing for adaptation into a commercial product, not just for its simplicity (*i.e.* scalability) and speed, but also for its use of cost-effective materials and ease of integration as a single step in POC bioassays that seek to use blood as a sample type but aim to separate plasma before performing an assay to simplify measurement.

A consideration for introducing microfluidic systems into commercial products is the challenge of sequential fluidics. It is often beneficial to achieve sequential introduction of reagents in an assay, with pre-determined times between each reagent, but academic research often benefits from having a trained operator to enact each step by pipetting after the passing of the prescribed incubation duration. In the ideal POCT, the operator would not perform many steps other than pressing start after applying the sample, and so the problem of introducing reagents must be resolved within the device itself. There have been several methods of sequentially introducing reagents in literature, such as the use of seals that can mechanically break (*e.g. via* heat for melting wax plugs or piercing seals to relieve vacuum), manipulation of trapped air within the strip, or use of variable channel lengths to delay fluid arrival, to name a few.^147,148^ Making use of microfluidics and instrumentation to enable sequential reagent introduction in POCT can simplify and standardise test processes through automation of actions, thereby expanding the range of potential end-users, and increasing the marketability and potential versatility.

Regardless of the methods used, the ability to robustly compare the performance of distinct techniques to highlight key attributes or improvements will facilitate the adoption of these methods into product designs. Torres-Castro *et al.* provided a comparison of many current high throughput blood separation techniques, but, importantly, highlighted the lack of standardisation of reported metrics.^149^ In their review, it was suggested that many research articles presenting new developments and technologies only report some of what they call ‘key merits’ – these being input/output concentrations, device efficiency, output purity, flow rate, and throughput (Table 4). The argument given is that only reporting some of these metrics will give half the picture, making direct and meaningful comparison difficult or impossible. Therefore, standardisation of reporting metrics would benefit not only academic research, but commercialisation efforts too. Additional considerations for industrial translation include performance under varied environmental conditions (*e.g*. humidity and temperature), batch-to-batch reproducibility, and long-term stability to support extended shelf life for global distribution.

**Table 4:** Key merits for evaluation of microfluidic performance. Information gathered from ref. 149

Metric	Description
Input concentration	Initial concentration of analyte or sample introduced into the device
Output concentration	Final concentration of the target analyte after processing
Device efficiency	Percentage of target analyte recovered or processed correctly
Output purity	Purity of the separated or processed product relative to contaminants
Flow rate	Volume of sample processed per unit time (*e.g.*, μL min^−1^)
Throughput	Total volume or number of samples processed over a specified period

## Conclusions

As the COVID-19 pandemic demonstrated and the burden on healthcare systems grows, the need for innovative, low-cost, commercially available diagnostics that meet the REASSURED criteria is more apparent than ever. The paper describes how research groups can imbue their biosensor projects with ‘design thinking’ to maximise their chances of achieving commercial translation. Key factors outlined include identifying user needs, defining a suitable target product profile, and using these to inform device design and the necessary performance requirements. Recent important technical advances in crucial elements of biosensor development are summarised, including biorecognition elements, electrochemical measurements, materials selection and use of microfluidics. Currently, academic biosensor research is a vibrant growing field with a range of new and compelling sensor demonstrations being published frequently. It is unprecedented that the route to translation and eventual uptake of a product is so accessible to academic teams. Their tendency to form links with clinical teams (gaining access to samples), the ease of access to additive manufacturing technologies, working with SMEs and the current interest from potential investors in MedTech opportunities all equate to their huge potential for achieving translation.

## Author contributions

Authorship contributions are tracked using the appropriate CRediT system categories: conceptualisation – DC, ND; funding acquisition – DC, MJ; visualisation – ND, VM; writing – original draft – ND, DM, AG, AD, VM, DC; writing – review & editing – ND, DM, AG, AD, VM, DC, SP, YF, MJ.

## Conflicts of interest

There are no conflicts to declare.

## Data Availability

No primary research results, software or code have been included, and no new data were generated or analysed as part of this review.
